# PERFECTED enhanced recovery (PERFECT-ER) care versus standard acute care for patients admitted to acute settings with hip fracture identified as experiencing confusion: study protocol for a feasibility cluster randomized controlled trial

**DOI:** 10.1186/s13063-017-2303-y

**Published:** 2017-12-04

**Authors:** Simon P. Hammond, Jane L. Cross, Lee Shepstone, Tamara Backhouse, Catherine Henderson, Fiona Poland, Erika Sims, Alasdair MacLullich, Bridget Penhale, Robert Howard, Nigel Lambert, Anna Varley, Toby O. Smith, Opinder Sahota, Simon Donell, Martyn Patel, Clive Ballard, John Young, Martin Knapp, Stephen Jackson, Justin Waring, Nick Leavey, Gregory Howard, Chris Fox

**Affiliations:** 1 Department of Psychological Sciences, Norwich Medical School, Faculty of Medicine and Health Sciences, University of East Anglia, Norwich Research Park, Norwich, NR4 7TJ UK; 2 School of Health Sciences, Faculty of Medicine and Health Sciences, University of East Anglia, Norwich Research Park, Norwich, NR4 7TJ UK; 3 Norwich Medical School, Faculty of Medicine and Health Sciences, University of East Anglia, Norwich Research Park, Norwich, NR4 7TJ UK; 4 Department of Social Policy, London School of Economics and Political Science, Houghton Street, London, WC2A 2AE UK; 5 Norwich Clinical Trial Unit, Norwich Medical School, Faculty of Medicine and Health Sciences, University of East Anglia, Norwich Research Park, Norwich, NR4 7TJ UK; 6 Geriatric Medicine, University of Edinburgh, Edinburgh, EH16 4SA UK; 7 Division of Psychiatry, Faculty of Brain Sciences, University College London, London, W1T 7NF UK; 8 Nottingham University Hospitals NHS Trust, Queens Medical Centre Campus, Derby Road, Nottingham, NG7 2UH UK; 9 Norfolk and Norwich University Hospitals NHS Foundation Trust, Colney Lane, Norwich, NR4 7UY UK; 10 Exeter Medical School, University of Exeter, Penryn Campus, Penryn, Cornwall, TR10 9FE UK; 11 Faculty of Medicine and Health, University of Leeds, Leeds, LS2 9JT UK; 12 Department of Clinical Gerontology, King’s College Hospital NHS Foundation Trust, Denmark Hill, London, SE5 9RS UK; 13 Nottingham University Business School, Jubilee Campus, Nottingham, NG8 1BB UK

**Keywords:** Dementia, Hip fracture, Feasibility, Acute care, Hospital, Service improvement

## Abstract

**Background:**

Health and social care provision for an ageing population is a global priority. Provision for those with dementia and hip fracture has specific and growing importance. Older people who break their hip are recognised as exceptionally vulnerable to experiencing confusion (including but not exclusively, dementia and/or delirium and/or cognitive impairment(s)) before, during or after acute admissions. Older people experiencing hip fracture and confusion risk serious complications, linked to delayed recovery and higher mortality post-operatively. Specific care pathways acknowledging the differences in patient presentation and care needs are proposed to improve clinical and process outcomes.

**Methods:**

This protocol describes a multi-centre, feasibility, cluster-randomised, controlled trial (CRCT) to be undertaken across ten National Health Service hospital trusts in the UK. The trial will explore the feasibility of undertaking a CRCT comparing the multicomponent PERFECTED enhanced recovery intervention (PERFECT-ER), which acknowledges the differences in care needs of confused older patients experiencing hip fracture, with standard care. The trial will also have an integrated process evaluation to explore how PERFECT-ER is implemented and interacts with the local context. The study will recruit 400 hip fracture patients identified as experiencing confusion and will also recruit “suitable informants” (individuals in regular contact with participants who will complete proxy measures). We will also recruit NHS professionals for the process evaluation. This mixed methods design will produce data to inform a definitive evaluation of the intervention via a large-scale pragmatic randomised controlled trial (RCT).

**Discussion:**

The trial will provide a preliminary estimate of potential efficacy of PERFECT-ER versus standard care; assess service delivery variation, inform primary and secondary outcome selection, generate estimates of recruitment and retention rates, data collection difficulties, and completeness of outcome data and provide an indication of potential economic benefits. The process evaluation will enhance knowledge of implementation delivery and receipt.

**Trial registration:**

ISRCTN, 99336264. Registered on 5 September 2016.

**Electronic supplementary material:**

The online version of this article (doi:10.1186/s13063-017-2303-y) contains supplementary material, which is available to authorized users.

## Background

Hip fracture is one of the commonest orthopaedic injuries [1]. It is estimated that more than 1.2 million individuals worldwide suffer hip fracture annually [2], a total expected to surpass 6 million by the year 2050 [1]. Hip fracture has a substantial impact on the health, wellbeing and independence of patients and their families. It also generates high costs for health and social care systems; for example the impact on the National Health Service (NHS) in the UK is estimated at £2 billion per annum [3]. In the first 12 months post-fracture, patients are at increased risk of functional decline, admission to long-term care institutions and higher mortality rates [4].

Over 40% of people with hip fracture have dementia or cognitive impairment [5]. This can impact on rehabilitation and recovery following hip surgery [5, 6]. Pre-existing “confusion” (including, but not exclusively, dementia and/or delirium) has been associated with greater risk of postoperative complications such as pressure ulcers and chest infections [4]. People who are confused may also find it more difficult to express pain and discomfort, making the assessment of these conditions more challenging [6]. The risk of confusion is of particular concern for people admitted to acute setting with prior memory difficulties and can have a more serious impact on their daily life [7, 8].

Confusion can compromise a patient’s ability to engage and progress with postoperative hip fracture rehabilitation. There is currently little evidence to guide practitioners to choose the care and rehabilitation interventions that will achieve the best outcomes for confused older people [9]. This has led to calls for patients with hip fracture experiencing confusion to have a specific treatment pathway, to acknowledge the differences in presentation and care needs.

A specified process of “enhanced recovery” has been used in UK health services since the early 2000s [10]. Enhanced recovery pathways (ERP) are becoming standard practice for many elective surgical operations [11]. Enhanced recovery practices rely upon integrated, multi-modal, evidence-based approaches that aim to enable patients to recover and leave hospital sooner after surgery. Whilst embedded in many elective practices, it has not been standard to take an enhanced recovery approach in treating acute hip fracture.

The National Hip Fracture Database (NHFD) was developed and launched by the Royal College of Physicians as part of their “Falls and Fragility Fracture programme” [12]. The NHFD is web-based and requires data to be entered on auditable aspects of the care delivered to every patient with hip fracture across Northern Ireland, Wales and England. It integrates nationally agreed standards for the management of patients aged 60 years or older as set by the British Orthopaedic Association, British Geriatrics Society and National Institute for Health and Care Excellence (NICE) clinical guideline. NHFD data support a system of “payment by results” in England and Wales known as the Best Practice Tariff (BPT). Findings on the impact of the NHFD and BPT have demonstrated improved outcomes via the co-management of patients by consultant geriatrician and orthopaedic surgeon [13], a reduction in mortality rates [14] and decreased time to surgery [15]. Although not delineated in relation to how care could/should be delivered to patients with hip fracture experiencing confusion, these studies do suggest that multicomponent care interventions can improve a range of patient and clinical outcomes. Internationally, the picture is similar. Interventions show potential but do not provide guidance on the best methods of delivering care to older confused patients with hip fracture [16–19]. Hence, answers to core questions remain relating to the best types of preoperative, postoperative and rehabilitation approaches for hip fracture care for patients experiencing confusion, and how such pathways can be delivered.

To address this global priority, a multicomponent intervention was developed as part of the “Peri-operative enhanced recovery hip fracture care of patients with dementia” (PERFECTED) research programme. PERFECTED encompassed several iterative and sequential sub-studies (“Care delivery in acute hospital settings: an observational study” UKCRN ID 16998, REC: 14/EM/1020, “Caring for patients with hip fracture and memory difficulties” (UKCRN ID 18281, REC: 15/EE/0007) and “Implementing the optimisation of hospital care delivery to older adults by NHS staff via action research methodology” (UKCRN ID 179797, REC: 15/SC/0294)). The PERFECTED enhanced recovery intervention (henceforth PERFECT-ER) was developed in accordance with the Medical Research Council (MRC) framework for complex interventions [20, 21] and aims to enhance recovery of patients with hip fracture experiencing confusion (including presumed dementia and/or other cognitive impairments such as delirium). The objective of the current study is to explore potential efficacy and cost-effectiveness of PERFECT-ER versus standard care in older patients with hip fracture who are experiencing confusion. The study’s ultimate objective is to inform a definitive evaluation of PERFECT-ER via a large-scale pragmatic randomised controlled trial (RCT). The protocol format is guided by the Standard Protocol Items: Recommendations for Interventional Trials (SPIRIT) checklist (Additional file 1) and the Consolidated Standards of Reporting Trials (CONSORT) extension to cluster-randomized trials [22, 23].

## Methods

### Aims and objectives

The aim of this study is to test trial and intervention feasibility to inform a definitive trial. The current study therefore has the following research objectives:

1. Generate preliminary estimates of potential efficacy of the PERFECT-ER versus standard care at 1, 3 and 6 months postoperatively
2. Assess service delivery variation between control and active wards
3. Inform primary and secondary outcome selection
4. Generate estimates of recruitment and retention rates, data collection difficulties, and completeness of outcome and costs data
5. Estimate the intra-class correlation coefficients for efficacy endpoints in order to inform sample size calculations for a later study
6. Assess potential intervention effectiveness
7. Assess the resource use, costs and outcomes associated with the intervention
8. Collect data to inform a comprehensive process evaluation

### Design

**Fig. 1:**
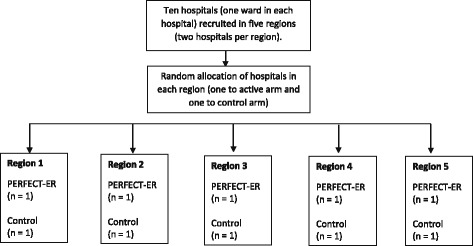
Randomisation overview; we will recruit ten hospitals across five separate geographical UK regions. One hospital per region will be randomised to active or control arm

This multicentre, interventional, feasibility study is an open trial, which will be cluster randomised. In line with MRC guidance for complex interventions [20, 21], an integrated multimethod multi-perspective (patients, suitable informants (defined as individuals with regular contact who are prepared to complete proxy measures) and NHS professionals) process evaluation will be conducted. This will explore variations in implementation fidelity, dose and reach. Trial hospitals will be randomised to active or control arm within geographical region using a simple randomisation process. An ad hoc programme will be written in SAS to carry out this procedure (see Fig. 1 Randomization overview). The nature of PERFECT-ER means that patients, suitable informants and staff delivering treatments cannot be masked to the trial arm. Accordingly, this is an open unblinded trial. Statistical analysis will be undertaken by a subgroup-blind approach, blinded to trial arm and cluster.

### Setting

The study will be conducted in acute hospital wards in ten NHS hospitals located in five different UK regions (England: four regions, eight hospitals; Scotland: one region, two hospitals) to which individuals who sustain proximal hip fracture are admitted. Each hospital will contribute one study ward. The unit of randomisation is the cluster, in this case, the hospital site. The following site inclusion and exclusion criteria have been identified.

Site inclusion criteria:

- Sites have an average monthly admission of at least 12 individuals who sustain proximal hip fracture requiring an operation and have a preoperative Abbreviated Mental Test Score (AMTS) ≤8 (England) or a 4AT ≥1 (Scotland) in the last 12 available calendar months
- Sites are able to provide the PERFECTED trial team with contextual ward level data (comprising BPT scores, number of falls, pressure ulcers, deaths and safeguarding incidents) in the last 12 available calendar months

Site exclusion criteria: sites that have participated in the “PERFECTED WP2: Implementing optimised hospital care” research programme leading to the development and refinement of PERFECT-ER will be excluded.

### Participants

Because of the cognitively vulnerable nature of the patient population, proxy measures will be used alongside patient-generated measures. Consent will therefore be sought from patients where possible and from “suitable informants” as defined subsequently. The eligibility criteria for patient participants and suitable informant participants are as follows.

Patient inclusion criteria:

1. Patient must have had confirmed proximal hip fracture requiring an operation and be aged ≥ 60 years at the time of the operation
2. Patient has a preoperative AMTS ≤8 in England (including those with zero because of an inability to answer questions) or a 4AT score ≥1 in Scotland
3. Patient must have a “suitable informant” (e.g. relative, unpaid or paid carer, care home manager) who has a minimum of once a month face-to-face or telephone contact with the patient and is able, and consents to, provide information on proxy measures
4. Patient and a suitable informant must be recruited into the trial within 7 days of the hip fracture operation
5. Patient must spend a minimum of 5 days on the study ward

Patient exclusion criteria:

1. Decision taken not to undergo hip surgery
2. Patient not expected to survive beyond 4 weeks
3. Patient already enrolled in a clinical trial of an investigational medicinal product (CTIMP)

Suitable informant inclusion criteria:

1. Individual has a minimum of once a month face-to-face or telephone contact with the patient
2. Individual is able, and consents to provide information on proxy measures

Suitable informant exclusion criteria: Individual not over 16 years of age.

### Intervention

PERFECT-ER is a multicomponent service improvement intervention with a systematic implementation process. It comprises of the PERFECT-ER checklist, which synthesizes best practice evidence of hospital-based dementia care delivery with current best practice evidence of the admission, preoperative, postoperative, rehabilitation and discharge management of hip fractures, agents of change (described below) and a model for change (plan-do-study-act). The checklist, consisting of patient-level and organizational-level care elements, is designed to overlay existing hip fracture pathways highlighting areas to improve care delivery to patients experiencing confusion. PERFECT-ER has been developed, piloted and refined in earlier PERFECTED work packages in partnership with a range of stakeholders in diverse development sites. As the current paper is reporting the protocol for a feasibility study the intervention is understandably not yet in the public domain.

Sites allocated to the active arm will be given PERFECT-ER 3 months before recruitment begins and resourced to support the implementation of PERFECT-ER using the NHS Service Improvement (plan-do-study-act) methodology [24]. The resource is in the form of an internal chance agent, known in this study as a Service Improvement Lead (SIL). Following the 3-month implementation period led by the SIL (and provided that individual active sites have reached predesignated PERFECT-ER adherence scores) sites will open to recruitment.

### Control arm

Control is treatment as usual. Local practices in each site will differ. We will collect relevant site profile data (please see “Site profile data” for details).

### Recruitment and consent procedures

Recruitment will commence when individual active sites have reached predesignated PERFECT-ER adherence scores. Recruitment will take place over a 10-month period; participants will be followed for 6 months. A three-step recruitment process has been guided by our experiences from previous phases of the PERFECTED programme, other studies in this area [25, 26] and conversations with clinical and academic collaborators:

1. Research nurses will collaborate with relevant clinical staff (including the study ward trauma co-ordinators and key Emergency Department colleagues) to identify all new hip fracture admissions and screen for pre-recruitment eligibility
2. Each patient (and where possible their potential suitable informant) will be approached by a research nurse who provide them information about the study as soon as clinically appropriate. During this initial approach the research nurse will assess the mental capacity of the patient.
3. The research nurse will approach the patient (where possible) and identified suitable informant to obtain informed consent. In England, where a patient is assessed as lacking capacity, personal or nominated consultee advice about inclusion will be sought. In Scotland, where a patient is assessed as lacking capacity, a welfare guardian, welfare attorney or nearest relative (henceforth known as a legal representative) will be sought and asked to consent in relation to the patient’s participation in the study. If appropriate and willing, suitable informants can also be personal consultees (England) or legal representatives (Scotland).

**Fig. 2:**
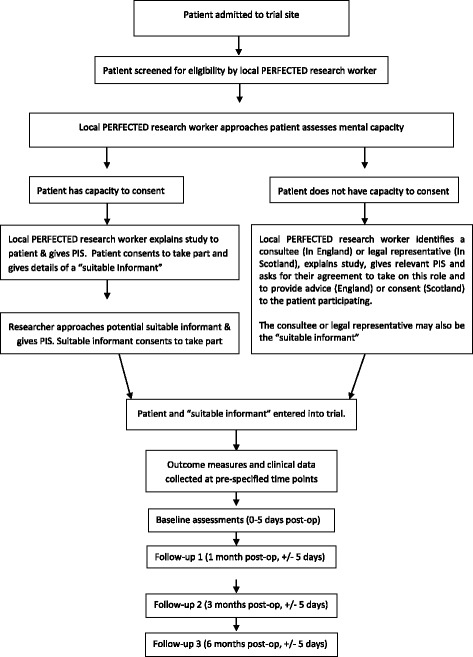
Recruitment overview; recruitment will take place over a 10-month period with a 6-month follow-up period. All patients entering study wards will be screened for eligibility. Local research workers will assess patient capacity. Patients interested in participating and assessed as having capacity will be consented into the study. Patients assessed as not having capacity will be able to take part in line with legislative guidance for individuals lacking capacity

This three-step process will be closely monitored by the coordinating centre to identify trends that might be leading to over-recruitment or under-recruitment from specific groups. For example, if sites are consenting purely via personal consultees (England) or legal representatives (Scotland), monitoring will enable corrective actions and provide information to mitigate these recruitment trends in any future definitive trial. A more detailed overview of the recruitment process is shown in Fig. 2.

### Recruiting patients with fluctuating and/or reduced capacity in England and Scotland

The aims of this trial are incompatible with only enrolling patients with minimal or mild confusion. It is important to ensure findings are broadly applicable and able to inform a future definitive trial. Participants who lack capacity to provide informed consent must be included. In this situation, the patient’s agreement to participate will still be obtained to their best level of understanding (in line with legislative frameworks in England and Scotland). Where patients in England are assessed as lacking capacity to make a decision about their initial or continued involvement with the study, we will seek personal or nominated consultee advice [27]. In Scottish trial sites where a patient is assessed not to have capacity, a legal representative will be sought and asked to consent in relation to the patient’s participation in the research [28]. In cases where written informed consent cannot be obtained, verbal consent can be taken (for example a patient with extremely poor eyesight or wrist/arm fractures). However this must be witnessed and fully documented in the patient’s notes [29]. For suitable informants in instances where written informed consent cannot be obtained, verbal consent can be taken over the telephone but must be witnessed and fully documented in the patient’s notes [29].

### Data collection

**Fig. 3:**
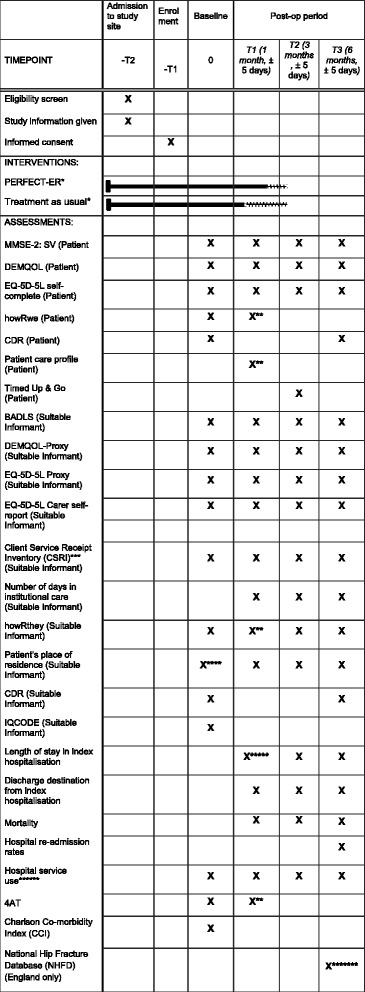
Detailed assessment. MMSE, Mini-Mental State Examination; EQ-5D-EL, EuroQol Five Dimensions Five Levels questionnaire; DEMQOL, Dementia Quality of Life; *PERFECT-ER and treatment as usual continue up until discharge from study ward. Due to differences in length of stay in the study sites, T1 assessments may take place in the study site for some participants; **Patients may be discharged from study ward before or after T1. Measure to be collected at whenever this point maybe ± five days; ***duration of retrospective period covered varies by assessment point; ****pre-baseline ordinary residence; *****If patient is still in acute hospital at thirty days this will be recorded; ******from hospital patient records, of service use within site of index hospitalisation; *******extracted from NHFD post recruitment window closing

This feasibility study will not have a primary outcome. Instead, this study will inform the selection of the primary outcome for a potential definitive evaluation of PERFECT-ER. Because of the complexity and feasibility nature of this trial, different types of data will be collected from different sources (see Fig. 3). Additional information about selected outcome measures detailed in Fig. 3 can be found in Additional file 2.

All outcome assessment timeframes are taken from the point after a patient returns from surgery. Local research nurses will collect data from patients and their suitable informants at four time points. Assessment timeframes are baseline (0–5 days postoperative), follow up at time 1 (1 month ± 5 days), time 2 (3 months ± 5 days), and time 3 (6 months ± 5 days). Follow-up measures will be obtained by a local research nurse at the patient’s place of residence, with suitable informant follow-up data collected according to pragmatic arrangements.

We will also collect clinical measures and resource use data (Fig. 3). We will collect hospital care data (Accident and Emergency visits, inpatient and day-case admissions, outpatient appointments) from the clinical records of the participating NHS Trusts. Data on health and social care services and unpaid carers used by patients will be collected at each follow-up time point from the suitable informants (however only informants who are unpaid carers will be asked to complete questions on unpaid carer input). This will allow comparison of hospital-service use data from both sources. Findings from this collection and from the process evaluation workstream will also be used to explore the feasibility of collecting hospital service-use and cost data from routine sources. For patients cared for in English trusts, we will also collect data made available via the NHFD. Findings will also be used to explore the feasibility of economic data collection. We will interrogate these data to test for differences between participating hospitals to inform later generalisability. We will also draw on these results to make recommendations for cost data-collection methods for a definitive trial if indicated, taking into account research costs, data completeness and reporting burden experienced by participants.

### Site profile data

To anticipate the expected wide variance in local practices, sites will complete a ward profiling assessment (comprising hospital characteristics, study ward characteristics, study ward staff profiling, and patient care profiles at organisational and patient level) at baseline, 4, 7 and 10 months following the trial opening. This will enable any drift from baseline status towards perceived improvements in service delivery to be monitored across control and active arms and provide rich contextual data for the process evaluation.

### Process evaluation data

Following MRC guidance for complex interventions [21], an integrated multimethod, multi-perspective (patients, suitable informants and NHS professionals) process evaluation will be conducted. The process evaluation will aim to determine how, and under what circumstances, PERFECT-ER is implemented in each site and to examine how, and under what circumstances, implementing PERFECT-ER impacts on staff practices and perceptions, resource use, and patient and suitable informant outcomes. Learning from initial piloting of PERFECT-ER and its implementation mechanisms (PERFECTED WP2: Implementing the optimisation of hospital care delivery to older adults by NHS staff via action research methodology) will be used to inform procedures for collecting quantitative and qualitative data to inform the process evaluation. This data set will comprise PERFECT-ER checklist scores and SIL-generated reports and action plans. We will also undertake semi-structured interviews with patients (where appropriate) and suitable informants with experience of PERFECT-ER care delivery (25 patients and 25 suitable informants, 5 per population per active site). Interviews with patients will take place at their place of residence at the time (hospital, rehabilitation unit, care home or patient’s home). Locations and mode (face-to-face or telephone) of semi-structured interviews with suitable informants will be organised on a case-by-case basis sensitive to individual preference. Public patient involvement (PPI) is a central part of the PERFECTED programme. PPI colleagues are experts by experience [30]. They will receive training and assist in co-interviewing of suitable informants alongside academic researchers on a case-by-case basis.

Additionally, interviews and focus groups will be held with NHS professionals involved in ward-level and strategic implementation of PERFECT-ER. This research will also explore the economic aspects of caring for people who are confused and have hip fracture (for instance, to understand how time is used to provide appropriate care to this patient group). In addition, we will look at the feasibility of collecting detailed patient-level costing information (e.g. patient-specific nursing time, laboratory tests) from administrative systems in each site, mapping the availability of information based on correspondence with hospital administrators/finance officers and a documentary analysis.

### Sample size

The target sample size is 400 patient participants (200 per arm). This will comprise 40 patient participants’ in 10 different sites. As this is a feasibility study, this sample size was not decided on the basis of efficacy analyses; rather this is a purposive selection of groups with relevant experience of hip fracture and confusion and ability to access in the time available. Their data will be used to estimate the intra-class correlation coefficient for each outcome measure. Assuming a coefficient of no more than 0.1, 400 participants from 10 centres should provide a standard error no greater than 0.041, providing a basis for a definitive trial sample size.

## Analysis

### Quantitative analysis

Data from the trial will be analysed and reported in accordance with Consolidated Standards of Reporting Trials (CONSORT) [23]. An initial estimate of intervention efficacy will be provided using a multi-level model, with hospital as the highest “cluster” level and an appropriate error term depending on the nature of the outcome of interest. Between-treatment-arm effects will be estimated with 95% confidence intervals. Analyses will be on an intention-to-treat basis. There are no plans for imputing missing data and thus analyses will be on a complete-case basis only. No subgroup analyses are planned. In the light of new information, should it be decided during the course of the trial that a subgroup analysis is appropriate, this will be recorded in the statistical analysis plan prior to any data analyses. There will be no formal interim analysis. Deaths, falls, pressure ulcers, admissions to long-term residential care and safeguarding incidents are expected in this patient population. These rates will be collected at ward level throughout the study period. Analysis of recruitment rates, patient participant (including mortality) and suitable informant withdrawal rates will also be reviewed. Based on the data collected from this study, a formal sample size calculation will be carried out to inform a future, definitive trial.

Health and social care costs will be calculated drawing on the trial data, attaching unit costs from published sources to quantities of service use [31, 32]. The additional resource use associated with the intervention will be examined over the period of the trial by tracking potential indicators of impact, such as patient time spent in the anaesthetic room (i.e. this could change because relatives are encouraged to stay with the patient as part of the enhanced recovery checklist). SIL salary costs, on-costs and overheads will be estimated in order calculate their contribution to the costs of participants’ index hospital stays. Cost-effectiveness analyses will be from two perspectives - health and social care and societal. We will analyse outcomes including Bristol Activities for Daily Living Scale (BADLS) (patients) and quality-adjusted life-year (QALY) gain (for patients and carers). Incremental cost-effectiveness ratios will be computed and cost-effectiveness acceptability curves generated to examine the probability of cost-effectiveness over a range of willingness-to-pay thresholds for each outcome. Multivariate analyses of costs and outcomes appropriate for the clustered-randomised trial design will control for centre, baseline outcome measures or costs, and other confounders. We will compare results of analyses using societal, patient and carer weights for QALYs generated from DEMQOL-proxy, and different approaches to valuing unpaid care [32]. Variations in hospital service use within and between patients (e.g. hospital admissions, Accident and Emergency visits) will be explored through latent growth modelling. A sample of Participant Information Sheets and Consent Forms can be found in Additional file 3.

### Qualitative analysis

Data such as audio-files not already in a textual form will be transcribed verbatim and represented as “play script” transcripts. Once transcribed and formatted these documents will be imported into the NVivo qualitative software. Analysis will be conducted iteratively and draw out emergent themes from the data to explore variations in implementation fidelity, dose, adaptation and reach. The Consolidated Framework for Implementation Research (CFIR) will be used to further guide the analysis, including the identification of disconfirming data, points of implementation vulnerability will be examined to see how these may or may not lead to instances of breakdowns in the intervention and cases for critical learning that can inform the definitive trial will be taken forward [33]. Qualitative data collected pertaining to the association between costs and resource use, inputs and quality of care will also be explored. Data collected from mapping of patient-level hospital costing will be tabulated and summarised to draw out lessons for a definitive trial.

## Discussion

Hip fractures are one of the biggest challenges in the medical community, giving rise to an estimated financial burden in the UK alone of over £2 billion per annum [3]. This study will examine the feasibility, acceptability and economic consequences of a complex intervention aiming to optimise care delivery to older patients with hip fracture experiencing confusion. The study’s findings will be used to build evidence to propose a definitive evaluation of the intervention through a large-scale pragmatic RCT.

We have used the term “confusion” in order to reflect the real-world complexity of the acute hospital environment into which PERFECT-ER will be introduced. Informed by clinical knowledge and learning from the previous stages of our research, we note that PERFECT-ER may improve care delivery to patients with presumed dementia and other cognitive impairments such as delirium. For PERFECT-ER to make a positive impact on the care of older confused adults, it needs a broad approach. The majority of patients admitted with hip fracture who are experiencing confusion do not arrive with a confirmed dementia diagnosis. We will include confused patients (with known or presumed dementia and/or delirium and/or cognitive impairment(s)) of any severity, who incur a fractured hip, admitted from any setting. Presence of dementia and sub-type of dementia will be defined from medical notes at time-1 follow up.

The target patient group does present practical and operational challenges, particularly for gaining consent and accessing patients during follow up at their place of residence. Given the cognitive impairment of the patient participants, some outcome measures must be completed by proxy using suitable informants. We can anticipate a number of challenges. It may be difficult to locate suitable informants in a timely manner and to maintain contact with them over the follow-up period. An informant might be providing substantial support to a confused older person with hip fracture and could feel disinclined to participate in the research. Significant challenges to both evaluation and implementation may arise. The nature of the intervention is complex and the acute hospital ward environment into which PERFECT-ER will be introduced is high-pressured and ever-changing. Such challenges provide additional justification for the current feasibility study and multimethod, multi-perspective process evaluation. The findings will inform the development of a future large-scale pragmatic definitive RCT and help to guide the worldwide delivery of care to older patients experiencing hip fracture and confusion. Findings will be published in high-quality academic journals and at high-impact academic conferences. Dissemination to clinical and service user stakeholders will take many forms; these include via the programme’s social media channels and website and the investigators’ networks including those with local and national user groups.

## Trial status

This trial is currently ongoing and open to recruitment. Recruitment began on 21 November 2016 and will close on 31 January 2018.

## Additional files

Additional file 1: SPIRIT 2013 checklist: recommended items to address in a clinical trial protocol and related documents. (DOCX 34 kb)

Additional file 2: Additional information on outcome measures. (DOCX 24 kb)

Additional file 3: Sample Participant Information Sheet and Consent Form. (DOCX 983 kb)

## Abbreviations

4AT: Assessment tool for delirium and cognitive impairment (Alertness, Abbreviated mental test 4, Attention, Acute change or fluctuating course)
AMTS: Abbreviated Mental Test Score
BADLS: Bristol Activities for Daily Living Scale
BPT: Best Practice Tariff
CDR: Clinical Dementia Rating
CFIR: Consolidated Framework for Implementation Research
CONSORT: Consolidated Standards of Reporting Trials
CRCT: Cluster-randomised Control Trial
CSRI: Client Service Receipt Inventory
EQ-5D-5L: EuroQol Five Dimensions Five Levels questionnaire
ERP: Enhanced recovery pathways
IQCODE: Informant Questionnaire on Cognitive Decline
MMSE-2: SV: Mini-Mental State Examination-2: Standard Version
MRC: Medical Research Council
NHFD: National Hip Fracture Database
NHS: National Health Service
NICE: National Institute for Health and Care Excellence
NOF: Neck of femur
PERFECTED: Peri-operative Enhanced Recovery Hip Fracture Care of Patients with Dementia
PERFECT-ER: PERFECTED enhanced recovery intervention
PIS: Participant information sheet
QALY: Quality-adjusted life-year
SIL: Service Improvement Leads
SPIRIT: Standard Protocol Items: Recommendations for Interventional Trials
UK: United Kingdom
