# Supplementary material for: PERFECTED enhanced recovery (PERFECT-ER) care versus standard acute care for patients admitted to acute settings with hip fracture identified as experiencing confusion: study protocol for a feasibility cluster randomized controlled trial
Source: Trials. 2017 Dec 4;18:583. doi: 10.1186/s13063-017-2303-y (PMC5715500; doi:10.1186/s13063-017-2303-y)
Supplement: Supplementary file 2 — Additional information on outcome measures. (DOCX 24 kb) [file 13063_2017_2303_MOESM2_ESM.docx]

# Additional file 2: Figure S3. Additional information on outcome measures

| **Source of data** | **Domain** | **Instrument** | **Data collection time points** |
| --- | --- | --- | --- |
| Patient | Cognition | MMSE-2: SV [1] | Baseline, T1 (1 month, ± 5 days), T2 (3 months, ± 5 days), T3 (6 months, ± 5 days) |
| Patient | Quality of Life | DEMQOL [2] | Baseline, T1 (1 month, ± 5 days), T2 (3 months, ± 5 days), T3 (6 months, ± 5 days) |
| Patient | Quality of Life | EQ-5D-5L self-complete [3] | Baseline, T1 (1 month, ± 5 days), T2 (3 months, ± 5 days), T3 (6 months, ± 5 days) |
| Patient | Patient perspective on care | howRwe [4] | Baseline, T1* (1 month, ± 5 days) |
| Patient | Cognition | Clinical Dementia Rating (CDR) [5] | Baseline, T3 (6 months, ± 5 days) |
| Patient | Case note review tool, not yet in public domain | Patient care profile | T1* (1 month, ± 5 days) |
| Patient | Mobility | Timed Up & Go [6] | T2 (3 months, ± 5 days) |
| Suitable Informant | Physical function for activities for daily living | [Bristol Activities of Daily Living Scale](http://ageing.oxfordjournals.org/content/25/2/113.full.pdf) (BADLS) [7] | Baseline, T1 (1 month, ± 5 days), T2 (3 months, ± 5 days), T3 (6 months, ± 5 days) |
| Suitable Informant | Quality of Life | DEMQOL-Proxy [8] | Baseline, T1 (1 month, ± 5 days), T2 (3 months, ± 5 days), T3 (6 months, ± 5 days) |
| Suitable Informant | Quality of Life | EQ-5D-5L Carer self-report [3] | Baseline, T1 (1 month, ± 5 days), T2 (3 months, ± 5 days), T3 (6 months, ± 5 days) |
| **Source of data** | **Domain** | **Instrument** | **Data collection time points** |
| Suitable Informant | Service use | Client Service Receipt Inventory CSRI** [9] | Baseline, T1 (1 month, ± 5 days), T2 (3 months, ± 5 days), T3 (6 months, ± 5 days) |
| Suitable Informant | Service use | Number of days in institutional care | T1 (1 month, ± 5 days), T2 (3 months, ± 5 days), T3 (6 months, ± 5 days) |
| Suitable Informant | Carer perspective on patient care | howRthey [10] | Baseline, T1* (1 month, ± 5 days), T2 (3 months, ± 5 days), T3 (6 months, ± 5 days) |
| Suitable Informant | Service use | Patient’s place of residence question | Baseline***, T1 (1 month, ± 5 days), T2 (3 months, ± 5 days), T3 (6 months, ± 5 days) |
| Suitable Informant | Cognition | Clinical Dementia Rating (CDR) [5] | Baseline, T3 (6 months, ± 5 days) |
| Suitable Informant | Cognition | Informant Questionnaire on Cognitive Decline in the Elderly (IQCODE) [11] | Baseline only |
| Patient hospital records | Service use | Length of stay in index hospitalisation question | T1**** (1 month, ± 5 days), T2 (3 months, ± 5 days), T3 (6 months, ± 5 days) |
| Patient hospital records | Service use | Discharge destination from index hospitalisation question | T1 (1 month, ± 5 days), T2 (3 months, ± 5 days), T3 (6 months, ± 5 days) |
| Patient records | Hospital re-admission rates | Hospital re-admission rates question | T3 (6 months, ± 5 days) |
| Patient records | Hospital service use***** | Hospital service use question | Baseline, T1 (1 month, ± 5 days), T2 (3 months, ± 5 days), T3 (6 months, ± 5 days) |
| **Source of data** | **Domain** | **Instrument** | **Data collection time points** |
| Patient | Delirium | 4AT [12] | Baseline, T1* (1 month, ± 5 days) |
| Patient records | Comorbidity marker | Charlson Co-morbidity Index (CCI) [13] | Baseline, T1 (1 month, ± 5 days), T2 (3 months, ± 5 days), T3 (6 months, ± 5 days) |
| Patient records | Service delivery comparator | National Hip Fracture Database (NHFD) (England only) [14] | T3****** (6 months, ± 5 days) |

* Patients may be discharged from study ward before or after T1. Measure to be collected

whenever discharge occurs from study ward. This point maybe ± 5 days;

***duration of retrospective period covered varies by assessment point;

***pre-baseline ordinary residence;

**** If patient is still in acute hospital at 30 days this will be recorded;

*****from hospital patient records, of service use within site of index hospitalisation

******extracted from National Hip Fracture Database post recruitment window closing

# References

1. Folstein MF, Folstein SE, White T, Messer MA: **Mini-Mental State Examination, 2nd edition.** 2010. Psychological Assessment Resources, Florida.

2. Smith SC, Lamping DL, Banerjee S, Harwood R, Foley B, Smith P, et al: **Measurement of health-related quality of life for people with dementia: development of a new instrument (DEMQOL) and an evaluation of current methodology.** Health Technol Assess 2005, **9**(10).

3. Herdman M, Gudex C, Lloyd A, Janssen M, Kind P, Parkin D, Bonsel G, Badia X. **Development and preliminary testing of the new five-level version of EQ-5D (EQ-5D-5L).** *Qual Life Res*. 2011, **10:**1727-36.

4. Benson T, Potts HWW: **A short generic patient experience questionnaire: howRwe development and validation**. *BMC Health Services Research 2014*, **14**:499.

5. Morris JC. **The Clinical Dementia Rating (CDR): Current version and scoring rules.** *Neurology* 1993, **43**:2412-2414.

6. Podsiadlo D, Richardson S: **The timed "Up & Go": a test of basic functional mobility for frail elderly persons.** *J Am Geriatr Soc*. 1991, **2:** 142-8.

7. Bucks RS, Ashworth DL, Wilcock GK, Siegfried K: **Assessment of activities of daily living in dementia: development of the Bristol Activities of Daily Living Scale 1996.** *Age and Ageing,* **25:**113–120.

8. Smith SC, Murray J, Banerjee S, Foley B, Cook JC, Lamping DL, et al: **What constitutes health-related quality of life in dementia? Development of a conceptual framework for people with dementia and their carers.** *Int J Geriatr Psychiatry* 2005, **20**:889–895.

9. Beecham J, Knapp M: Costing Psychiatric Interventions, in Measuring Mental Health Needs, Eds.Thornicroft G, Brewin C & Wing JK. 1992. Gaskell: London.

10. **howRthey?**

[http://r-outcomes.com/measures/carer-measures/]

11. Jorm AF: **A short form of the Informant Questionnaire on Cognitive Decline in the Elderly (IQCODE): development and cross-validation**. *Psychol Med* 1994, **24**: 145–153.

12. Shenkin SD, Russ TC, Ryan TM, MacLullich AM: **Screening for dementia and other causes of cognitive impairment in general hospital in-patients**. *Age & Ageing* 2014, **43**:166-8.

13. de Groot V, Beckerman H, Lankhorst GJ, et al. **How to measure comorbidity: a critical review of available methods.** *J Clin Epidemiol.* 2003, **3:**221–229.

14. Royal College of Physicians. **National Hip Fracture Database annual report 2017**. London: RCP, 2017.
