# Supplementary material for: PERFECTED enhanced recovery (PERFECT-ER) care versus standard acute care for patients admitted to acute settings with hip fracture identified as experiencing confusion: study protocol for a feasibility cluster randomized controlled trial
Source: Trials. 2017 Dec 4;18:583. doi: 10.1186/s13063-017-2303-y (PMC5715500; doi:10.1186/s13063-017-2303-y)
Supplement: Supplementary file 3 — Sample Participant Information Sheet and Consent Form. (DOCX 983 kb) [file 13063_2017_2303_MOESM3_ESM.docx]

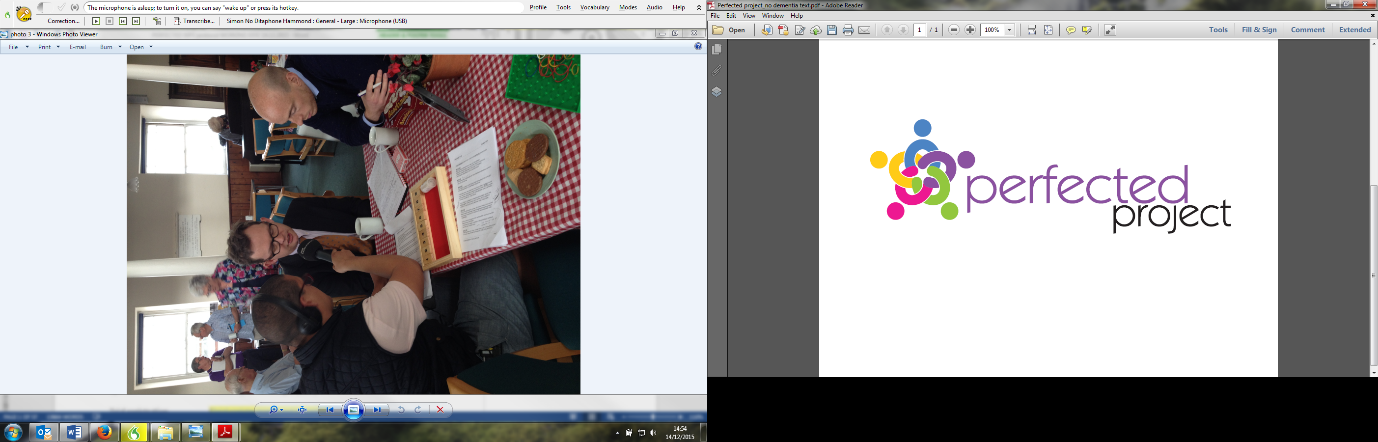

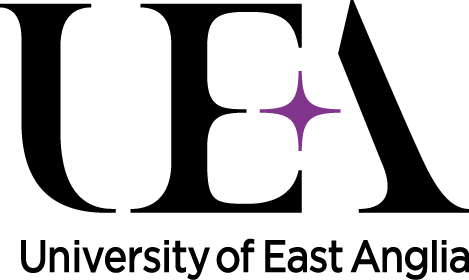


**Trial Participant Information Sheet: Patient**

**FULL VERSION**

**Care of patients experiencing hip fracture & confusion: PERFECTED**

Professor Chris Fox and members of his research team would like to invite you to take part in a research study into hip fracture and confusion. The study is called “PERFECTED”. You are eligible to take part in this study because you have fractured your hip and you have, or are experiencing confusion.

This leaflet tells you why the study is being done and provides details about what it would involve for you if you chose to take part. Please read it and discuss it with family, friends or carers.

We will also be asking you to name someone who you have contact with face-to-face or on the telephone at least once a month who we will ask to take part in this study.

**What is the purpose of this study?**

Hip fracture has a significant impact on the health and independence of patients and their families. Older people who fracture their hip can become confused whilst in hospital. For people with prior memory difficulties, the risk of confusion is greater, and can have a more serious impact on their daily life. The PERFECTED study aims to see if care of patients who fracture their hip and who experience confusion can be delivered more effectively. To do this we are comparing wards which use the learning from earlier parts of our PERFECTED study against wards delivering standard care.

**Why have I been chosen?**

You have been given this invitation because you are currently in a hospital taking part in this study and have fractured your hip. A member of your care team has suggested you as an eligible patient because you have experienced or are experiencing some confusion. We also believe that you are not taking part in any other research study at this time. Please inform us if this is not the case as it may mean you are not able to take part in this study.

# **Do I have to take part?**

No, it is up to you. If you prefer not to take part then please tell the person who approached you about this study. This will not affect the care that you receive in any way.

## What will happen to me if I take part?

We will ask you to sign a consent form stating that you agree to take part. We will access your medical and General Practitioner (GP) records to see how often you see NHS staff and how they help you. Whilst you are in hospital, we will ask you some questions about your memory and daily life.

At approximately 1, 3 and 6 months after your operation, a member of our research team will contact you to arrange a suitable time to visit you to ask you questions about what your memory and daily life have been like since your operation.

- When we visit you at 1 month after your operation, we may also invite you to take part in an interview about the care you received in this hospital. If selected we will give you more information about the interview at this visit.
- We are also interested in your mobility following your operation. At the 3 month visit we will ask you to stand up from a chair, walk 3 yards at your own speed and return to sitting in the chair. We will ask you to use any walking aids which you normally use during this task.

We realise that recovering from a major hip operation can be hard, particularly for those who have experienced some confusion. Because of this we would like you to tell us of someone in your life that you have contact with either face-to-face or via telephone at least once a month so we can ask them about your memory and daily life as well.

## Patients in England only

The National Hip Fracture Database uses care and treatment information collected by hospitals to look at the quality of care they provide. To help PERFECTED we wish to access the information collected by the National Hip Fracture Database. This information includes; the type of surgery you have, information about the surgery itself, what type of place you were living in before you broke your hip and if you went back to the same place following your operation. To do this we need your permission to record your NHS number. We will securely share this with the National Hip Fracture Database. They will remove your NHS number and send us the information we require confidentially.

**What happens if I become really confused?**

When some people experience confusion there may be occasions when they are unable to remember things and/or make informed choices. This is sometimes known as losing capacity.

If, during the study, you are assessed by a member of our research team to have lost capacity to make informed choices about your involvement with our study, we will seek advice from an appropriate person about your wishes. This person may be a family member or close friend. In England this person would become known as ‘consultee’, in Scotland this person would be known as a ‘legal representative’.

Speaking to a consultee or legal representative on your behalf will help our team to keep you involved in the research project. If this happened, your involvement in the study would continue unless you appeared to object or we are told otherwise by your consultee (England) or legal representative (Scotland). It may be helpful to tell a member of our research team who you might like to advise us on your behalf in respect of your involvement in this research study, should you lose capacity.

**Are there any risks in taking part?**

No. Taking part in this research study should not cause you any problems. You will simply be asked questions which we would like you to answer as best you can. The walking activity only asks you to do what you may already be doing.

# **Are there any benefits in taking part?**

There are no direct benefits but this research may help NHS staff members care for people in the future.

**What happens if I do not want to carry on in the study?**

You are free to stop taking part in the study at any time. We will ask you for a reason for stopping as it will help the study, but you do not have to give a reason. If you choose to take part but then change your mind, any information you have agreed to provide until that point will be kept by the study but no further information will be collected. Not continuing in this study will not affect your future care in any way.

# **What if there is a problem?**

If you have concerns about any aspect of the study, please feel free to discuss the matter with a member of the research team. If you remain unhappy please contact XXXXXX XXXXXXXXX (PERFECTED research worker), XXXX XXXX (Local Study Lead) or Prof Ken Laidlaw (Head of Clinical Psychology, Norwich Medical School, UEA) on 01603 593600. If you have more general concerns about the care you received in hospital please access the Patient Advice and Liaison Service (PALS) on 0800 073 0741 or you can visit the PALS website at: <http://www.pals.nhs.uk/>

**What happens when the study is finished?**

The results of this study will interest many different people. Findings will be published but no one will be able to tell it was you. Data will be stored securely at the hospital and at the University of East Anglia for at least 10 years after the study ends and then securely destroyed.

# **Will my taking part in the study be confidential?**

All the information we collect during the course of the research will be kept confidential and there are strict laws which safeguard your privacy at every stage in line with the Data Protection Act (1998). Study researchers will need to access your medical and General Practitioner (GP) records to carry out this research. We will give you a unique study code. This allows us to remove personally identifiable information. As PERFECTED has a large study team anonymised data may be shared securely between research organisations but only for research purposes.

You may also be approached to participate in other research studies conducted by the Chief Investigator for PERFECTED in the future. If you decide to take part in these, the research team may wish to access some of your data from this study. If this is the case, the Sponsor through the Chief Investigator will ensure that the other research complies with legal, data protection and ethical guidelines and that any data shared between the research studies remains anonymous.

To ensure that the study is being run correctly, we will ask your consent for responsible representatives from the Sponsor (University of East Anglia) and the NHS Institution to access your medical and General Practitioner (GP) records and data collected during the study, where it is relevant to you taking part in this research. The Sponsor (University of East Anglia) is responsible for overall management of the study and providing insurance and indemnity.

The Sponsor, the NHS and all researchers involved have a duty of confidentiality and nothing that could reveal your identity will be disclosed outside the research site and the co-ordinating centre (University of East Anglia). To ensure the study is running appropriately a selection of consent forms will be sent to the University of East Anglia from local sites via secure fax or by encrypted email from a secure NHS mail account. These forms, like all the other data you provide, will be kept for a minimum of 10 years after the study ends and then securely destroyed.

The only time that we would pass on identifiable information would be if you disclosed information of a serious incident or information that made us think that you or someone else, was at risk of serious harm. Additionally, if a member of the study team has reason to believe that you may be potentially suffering from depression we will send a letter to your General Practitioner (GP) informing them of this.

**Want to know more about the study?**

**PERFECTED** is funded by the National Institute for Health Research. This study and its documents have been reviewed and approved by a National Research Ethics Committee. This is an independent group of people who review research studies to protect the safety, rights, well-being and dignity of participants in research.

If you have any further questions, please contact one of the following:

- XXXXXXXXXXXXXXXXXXXX (PERFECTED research worker), address. Email: XXX, Tel: XXXXX XXXXXX
- XXXXXXXXXXXXX (Local Study Lead), Address, Email: XXX, Tel: XXXXX XXXXXX

# **Thank you for considering taking part in this research study and taking time to read this leaflet.**


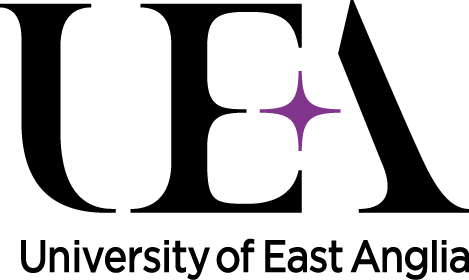

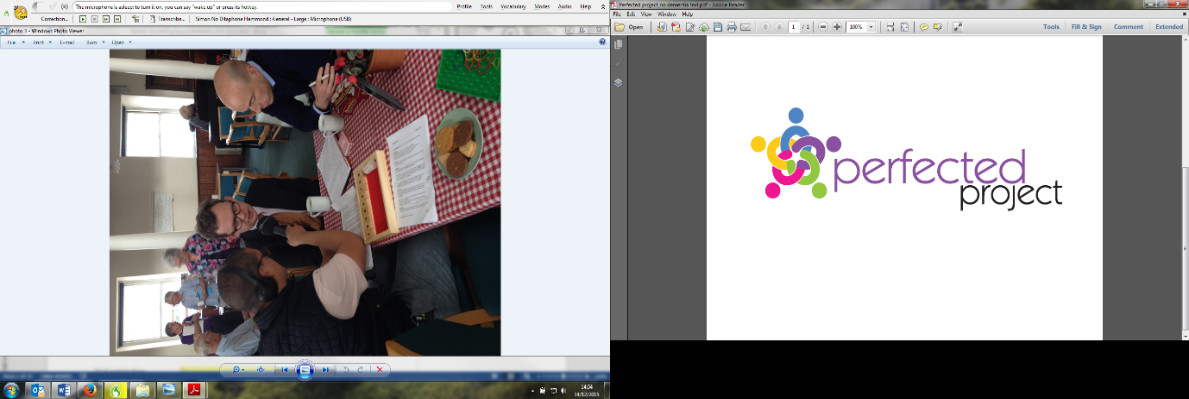


Trust Logo

**Trial Consent Form: Patient**

**Care of patients experiencing hip fracture & confusion: PERFECTED**

Chief Investigator: Professor Chris Fox

**Please initial box**

1. I have read and understand the information as specified in the Trial Participant Information Sheet: Patient, Version 4.0 (03.05.2017) and I have had the opportunity to consider the information, ask questions and have had these answered satisfactorily.

2. I agree to complete all study questionnaires to the best of my ability.

3. I agree that a member of the research team can access my medical and General Practitioner (GP) records in order to help with the study and that these records will be handled confidentially. I understand that (if I’m a patient of an English Trust) that this includes access to information collected for the National Hip Fracture Database accessed via the secure transfer of my NHS number.

4. I agree to undertake the walking task described to the best of my ability.

5. I understand that information will be anonymised, remain confidential, stored securely and be shared between research organisations for research purposes only.

6. I understand that to help ensure the project is being well run, monitors may review and/or transfer my information, and that this will be done in a confidential manner.

7. I understand that if the study team think I may be experiencing depression that they will inform my General Practitioner (GP) via letter.

8. I understand that I may be asked to take part in a research interview about this study and that further information will be given to allow me to make a decision around 1 month after my operation.

9. I understand that my participation is voluntary and that I am free to withdraw at any time without giving any reason and without any adverse consequences.

Participant number………………

10. I agree to take part in the study.

______________________ _______________ ___________________

Name of participant Date Signature

______________________ _______________ ___________________

Name of person taking consent Date Signature

When completed: 1 for participant; 1 for researcher site file; 1 for participant’s Medical Records.
